# 埃克替尼对比标准二线化疗治疗晚期非小细胞肺癌的临床观察

**DOI:** 10.3779/j.issn.1009-3419.2015.06.07

**Published:** 2015-06-20

**Authors:** 舒洋 姚, 坤 钱, 若天 王, 元博 李, 毅 张

**Affiliations:** 100053 北京，首都医科大学宣武医院胸外科 Department of Thoracic Surgery, Xuanwu Hospital Capital Medical University, Beijing 100053, China

**Keywords:** 肺肿瘤, 埃克替尼, 化疗, Lung neoplasms, Icotinib, Chemotherapy

## Abstract

**背景与目的:**

比较埃克替尼作为晚期非小细胞肺癌（non-small cell lung cancer, NSCLC）二线治疗药物与化疗的疗效及不良反应。

**方法:**

收集2012年1月-2013年7月首都医科大学宣武医院收治的32例接受盐酸埃克替尼作为二线药物以及33例同期接受化疗的晚期NSCLC患者的临床资料并进行回顾性分析。

**结果:**

埃克替尼组有效率（overall response rate, ORR）为28.1%，化疗组为18.2%，组间无统计学差异（*χ*^2^=0.905, *P*=0.341）。埃克替尼组疾病控制率为43.8%，化疗组为45.5%，组间无统计学差异（*χ*^2^=0.019, *P*=0.890）。埃克替尼组中，表皮生长因子受体（epidermal growth factor receptor, *EGFR*）突变者的ORR明显高于EGFR状态不明或阴性者（*χ*^2^=8.460, *P*=0.017）。埃克替尼中位无进展生存时间（progression-free survival, PFS）为4.1个月，化疗组为3.8个月，组间无明显差异（*P*=0.506）。通过*Cox*多因素回归分析，埃克替尼组患者的PFS与年龄、性别、病理类型以及一线最佳疗效无关。在不良反应的观察中发现埃克替尼不良反应发生率低于化疗组（*P*=0.001）。

**结论:**

与传统化疗相比，盐酸埃克替尼可作为治疗晚期NSCLC，尤其是EGFR基因状态未明的患者的有效药物，安全性更高，患者的耐受性更好。

## Abstract (en)

**Background and objective:**

This study compared the efficacy and safety of icotinib with standard second-line chemotherapy (single-agent docetaxel or pemetrexed) in previously treated advanced non-small cell lung cancer (NSCLC).

**Methods:**

Thirty-two consecutive patients treated with icotinib and 33 consecutive patients treated with standard second-line chemotherapy in Xuanwu Hospital from January 2012 to July 2013 were enrolled in our retrospective research. The Response Evaluation Criteria in Solid Tumors were used to evaluate the tumor responses, and the progression-free survival (PFS) was evaluated by *Kaplan-Meier* method.

**Results:**

Icotinib was comparable with standard second-line chemotherapy for advanced NSCLC in terms of overall response rate (ORR) (28.1% *vs* 18.2%, *P*=0.341), disease control rate (DFS)(43.8% *vs* 45.5%, *P*=0.890), and PFS (4.3 months *vs* 3.8 months, *P*=0.506). In the icotinib group, the ORR of epidermal growth factor receptor (*EGFR*) mutant was significantly higher than that of EGFR unknown or wild type (*P*=0.017). In multivariate analysis, age, gender, histology, and the optimum first-line treatment response were dependent prognostic factors based on the PFS of the icotinib group. The incidence of adverse events was significantly fewer in the icotinib group than in the chemotherapy group (*P*=0.001).

**Conclusion:**

Compared with the standard second-line chemotherapy, icotinib is active in the treatment of advanced NSCLC patients, especially with EGFR unknown in the second line, with an acceptable adverse event profile.

非小细胞肺癌（non-small cell lung cancer, NSCLC）仍是世界范围内发病率和死亡率最高的恶性肿瘤^[1]^。70%的NSCLC患者一经发现即为晚期，无法手术治疗，因此，药物治疗成为这部分患者延长生存时间改善生活质量的关键。然而，大部分晚期NSCLC患者在一线化疗4个周期后不久即出现疾病进展，而其中40%的患者在治疗过程中进展^[2]^。值得注意的是，约50%的一线治疗进展患者体力状态（performance status, PS）良好，能够耐受二线治疗^[3]^。目前，虽然多西他赛或培美曲塞等化疗药物被认为是PS评分好的患者的标准二线治疗，但是仍旧需要寻找更为有效及低毒的二线治疗方案^[4, 5]^。表皮生长因子受体（epidermal growth factor receptor, EGFR）酪氨酸激酶抑制剂（tyrosine kinase inhibitors, TKIs）的问世给晚期NSCLC患者带来福音，通过作用于EGFR酪氨酸激酶，阻断信号传导从而抑制肿瘤生长，延长了晚期肺癌患者的生存。由于口服用药，使用方便，不良反应轻，与标准二线化疗相比具有更好的耐受性^[6, 7]^。盐酸埃克替尼（商品名：凯美纳）作为继吉非替尼、厄洛替尼之后第三个在临床应用于晚期NSCLC的单靶点EGFR-TKI ^[8]^。Ⅲ期临床试验ICOGEN研究^[9]^已证实盐酸埃克替尼与吉非替尼在二、三线治疗NSCLC的疗效相当，埃克替尼的安全性更好。现对首都医科大学宣武医院2012年1月-2013年7月收治的32例接受盐酸埃克替尼以及33例同期接受化疗作为二线药物的NSCLC患者的临床资料进行回顾性分析，探讨疗效、安全性及影响因素。

## 1 材料与方法

### 1.1 研究对象

纳入标准：①首都医科大学宣武医院经病理或细胞学确诊的晚期NSCLC的患者；②影像学资料提示一线治疗中进展或治疗后复发；③一线治疗失败后至少接受1个月的埃克替尼或一周期的标准二线化疗。排除标准：①不适合接受化疗的患者；②预计生存时间小于3个月；③体能状态（performance status, PS）评分 > 2分；④血清总胆红素≥1.5倍正常值的患者；⑤中性粒细胞计数绝对值≤1, 500/μL的患者。

选取2012年1月-2013年7月首都医科大学宣武医院肺癌中心收治的晚期NSCLC患者65例。所有患者临床分期均为Ⅲb期或Ⅳ期（恶性胸腔积液、脑转移、肺转移、肝转移、骨转移等）。埃克替尼组32例，其中男性19例，女性13例，平均年龄为65.3（38-82）岁；化疗组33例，其中男性18例，女性15例，平均年龄为58.2（35-73）岁。埃克替尼组中腺癌29例，鳞癌3例；化疗组腺癌30例，鳞癌3例。*EGFR*基因突变状态，埃克替尼组突变3例，未突变11例，状态不明18例；化疗组突变3例，未突变者12例，状态不明者18例。一线治疗时，埃克替尼组有3例患者接受靶向治疗[吉非替尼3例，均为基因状态不明者，最佳疗效均为疾病稳定（stable disease, SD），无进展生存时间（progression free survival, PFS）分别为14个月、20个月和60个月]，29例接受化疗（吉西他滨联合卡铂14例，紫杉醇联合卡铂7例，多西他赛联合卡铂8例）；化疗组中有4例接受靶向治疗（吉非替尼2例均为*EGFR*突变者，最佳疗效均为SD，PFS分别为12个月和10个月；厄洛替尼2例均为基因状态不明者，最佳疗效1例为疾病进展（progressive disease, PD），另1例为SD，其PFS为5个月），29例接受化疗（吉西他滨联合卡铂13例，紫杉醇联合卡铂8例，多西他赛联合卡铂8例）。埃克替尼组吸烟者14例，非吸烟者18例；化疗组吸烟者17例，非吸烟者16例（表 1）。

**1:** 患者临床特征 Baseline characteristics of patients

Characteristics	Icotinib group (*n*=32)	Chemotherapy group (*n*=33)	*P*
Age (yr)			
< 65	15 (46.9%)	23 (69.7%)	
≥65	17 (53.1%)	10 (30.3%)	0.080
Gender			0.804
Male	19 (59.4%)	18 (54.5%)	
Female	13 (40.6%)	15 (45.5%)	
Histology			> 0.999
Adenocarcinoma	29 (90.6%)	30 (90.9%)	
Squamous	3 (9.4%)	3 (9.1%)	
Smoking			0.622
Yes	14 (43.8%)	17 (51.5%)	
No	18 (56.2%)	16 (49.5%)	
EGFR status			0.986
Mutation	3 (9.4%)	3 (9.1%)	
Wild-type	11 (34.4%)	12 (36.4%)	
Unknown	18 (56.2%)	18 (54.5%)	
First-line treatment			> 0.999
Chemotherapy	29 (90.6%)	29 (87.9%)	
Targeted therapy	3 (9.4%)	4 (12.1%)	
EGFR: epidermal growth factor receptor.			

### 1.2 治疗方法

埃克替尼组单服埃克替尼125 mg，口服每天3次，至PD或不良反应不能耐受时停药。化疗组将多西他赛75 mg/m^2^加入生理盐水250 mL中或培美曲塞500 mg/m2加入生理盐水100 mL中静滴，每周期均为21天，至疾病进展或不良反应不能耐受时停药。

### 1.3 观察指标及评价标准

临床近期疗效：埃克替尼组患者每月或因症状明显加剧进行计算机断层扫描（computed tomography, CT）、磁共振成像（magnetic resonance imaging, MRI）等相关影像学检查评价。化疗组患者一般每1个-2个周期化疗后进行CT、MRI等相关影像学检查以评定疗效。按照实体肿瘤疗效评价标准评价疗效，分为完全缓解（complete response, CR）、部分缓解（partial response, PR）、SD及PD。以CR+PR计算客观缓解率（overall response rate, ORR），CR+PR+SD≥6个月计算6个月疾病控制率（disease control rate, DCR)。药物不良反应：根据美国国立癌症研究所通用不良反应术语标准（National Cancer Institute Common Toxicity Criteria, NCI CTC）4.0版制定的药物不良反应进行分级。

### 1.4 随访

采用门诊、电话或书信方式随访，末次随访时间为2014年6月25日。PFS定义为埃克替尼或二线化疗开始至疾病进展或死亡的时间。

### 1.5 统计学方法

采用SPSS 17.0统计软件，两组间短期治疗效果的比较用卡方检验或*Fisher’s*精确检验；生存分析采用*Kaplan-Meier*法，多因素分析采用*Cox*多因素回归分析。*P* < 0.05为差异有统计学意义。

## 2 结果

### 2.1 近期疗效

埃克替尼组最佳疗效达PR 9例（28.1%），SD 13例（40.6%），PD 10例（31.3%）；化疗组患者疗效达PR 6例（18.2%），SD 16例（48.5%），PD 11例（33.3%），两组均无CR患者（表 2）。埃克替尼组ORR为28.1%，化疗组为18.2%，组间无统计学差异（*χ*^2^=0.905, *P*=0.341）。埃克替尼组6个月DCR为43.8%，化疗组为45.5%，组间无统计学差异（*χ*^2^=0.019, *P*=0.890）。埃克替尼组中，EGFR突变者（*n*=3）均达PR，EGFR状态不明或阴性者有6例达PR，突变者与状态不明或阴性者之间有统计学差异（*χ*^2^=8.460, *P*=0.017）。

**2:** 临床疗效的对比 Comparison of clinical efficacy in two arms

Group	*n*	CR	PR	SD	PD
Icotinib group	32	0 (0)	9 (28.1%)	13 (40.6%)	10 (31.3%)
Chemotherapy group	33	0 (0)	6 (18.2%)	16 (48.5%)	11 (33.3%)
CR: complete response; PR: partial response; SD: stable disease; PD: progressive disease.					

### 2.2 PFS

埃克替尼组中位PFS为4.1个月，化疗组为3.8个月，组间无明显差异（*P*=0.506）（图 1）。通过*Cox*多因素回归分析，埃克替尼组患者的PFS与年龄（*P*=0.169）、性别（*P*=0.531）、病理类型（*P*=0.371）以及一线最佳疗效（*P*=0.933）无关（表 3）。

**1:**
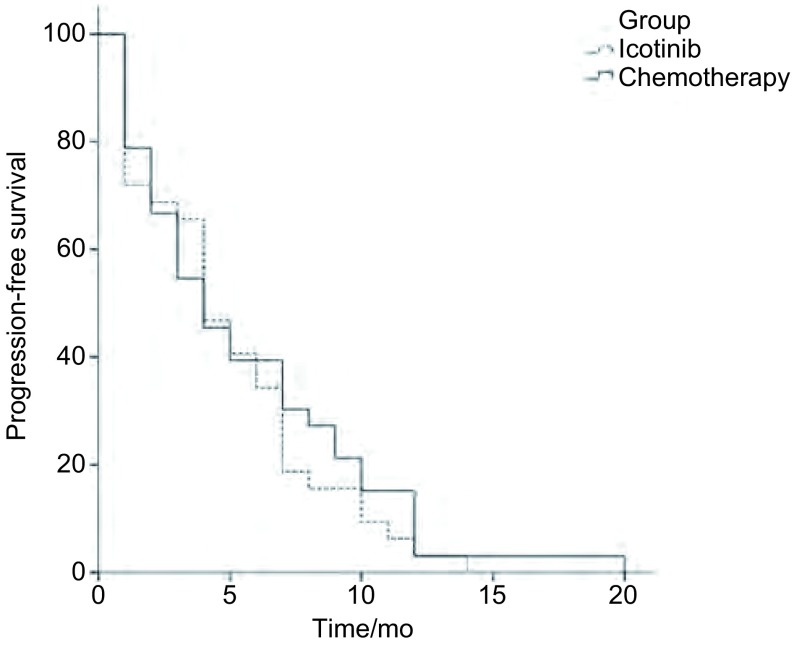
两组患者无进展生存时间的*Kaplan-Meier*曲线 *Kaplan-Meier* curve of progression-free survival for two arms

**3:** *Cox*多因素分析 Prognostic factors of PFS in *Cox* progression analysis

Variables	*β*	SE	*P*	HR	95%CI for HR
Age	-0.560	0.407	0.169	0.571	0.257-1.268
Gender	0.311	0.837	0.531	1.365	0.516-3.613
Histology	-0.638	0.713	0.371	0.528	0.130-2.138
1^st^-line best response	0.04	0.477	0.933	1.041	0.408-2.653
HR: hazard ratio.					

### 2.3 不良反应

埃克替尼组出现不良反应14例，大部分为Ⅰ度-Ⅱ度不良反应：皮疹（9例）、腹泻（3例）和肝功能损害（1例）。另有1例Ⅲ度腹泻。不良反应主要在治疗3周内出现，患者均能够耐受，无患者因不良反应终止治疗。化疗组主要不良反应为粒细胞减少、恶心、呕吐、脱发及肝功能损伤。化疗组3度及以上不良反应共9例：血液学毒性7例，胃肠反应1例，转氨酶升高1例。有2例患者因严重不良反应拒绝继续化疗。组间不良反应发生率差异明显（*χ*^2^=13.194, *P*=0.001）（表 4）。

**4:** 埃克替尼组与化疗组不良反应的比较 Adverse events of two arms

Group	Adverse events			*χ*^2^	*P*
	Without	Ⅰ-Ⅱ	Ⅲ-Ⅳ		
Icotinib group	18 (56.3%)	13 (40.6%)	1 (3.1%)	13.194	0.001
Chemotherapy group	6 (18.2%)	18 (54.5%)	9 (27.3%)		

## 3 讨论

多西他赛或培美曲塞单药是常用的治疗晚期NSCLC的二线化疗方案。通过包括INTREST^[6]^和TITAN^[7]^在内的多项前瞻性研究也已经证实吉非替尼和厄洛替尼是有效的治疗肺癌的二线药物。ICOGEN研究^[9]^结果说明一线或二线化疗失败的晚期NSCLC患者服用埃克替尼的疗效与吉非替尼相似，二者的ORR（27.6% *vs* 27.2%）、DCR（75.4% *vs* 74.9%）以及总生存时间（overall survival, OS）（13.3个月*vs* 13.9个月）均无统计学差异。与吉非替尼相比，埃克替尼组中位PFS更长（4.6个月*vs* 3.4个月，*P*=0.13，HR=0.84）。埃克替尼组的副作用如皮疹、腹泻和转氨酶升高的发生率明显低于吉非替尼组。因此，根据ICOGEN研究这一令人鼓舞的结果，2011年我国食品药品监督管理局批准埃克替尼用于二线或三线治疗晚期NSCLC。

两项比较EGFR-TKI单药与标准二线化疗治疗晚期NSCLC的荟萃分析^[10, 11]^发现，二者在OS、PFS和1年生存率方面无明显差异，而EGFR-TKI的ORR明显高于化疗组，不良反应明显低于化疗组，患者耐受性更好。国内有学者^[12]^对比了埃克替尼与多西他赛二线治疗晚期NSCLC患者，发现埃克替尼组DCR明显高于多西他赛组（83.8% *vs* 61.0%, *P*=0.025），而埃克替尼组PFS为2.6个月与多西他赛组的3.4个月相比，无统计学差异（*P*=0.419）。与这些研究结果相似，我们研究发现作为晚期NSCLC的二线治疗，与传统化疗药物相比，埃克替尼组ORR、DCR、PFS无明显差异。而本研究中DCR低于大部分研究，考虑原因为：①*EGFR*基因突变者仅为9%；②女性和非吸烟者所占比例不高，也就是说，EGFR基因状态不明者中突变率相对不高。在本研究的多因素分析中发现吸烟状态与PFS相关，而年龄、性别、病理类型、EGFR突变状态以及一线最佳疗效与PFS无关。本研究系回顾性分析，难以避免出现选择性偏倚。此外，这还可能与本研究中*EGFR*突变率低、EGFR-TKI治疗的优势人群所占比率小等因素有关。

不良反应方面，两组常见不良反应各异，埃克替尼组主要为皮疹、腹泻、肝转氨酶升高等；化疗组则以粒细胞减少、恶心、呕吐、脱发等较为常见，但总体上来说埃克替尼组不良反应发生率明显低于化疗组（*P*=0.001）。埃克替尼组没有患者因治疗相关性毒副作用停止治疗，而化疗组有2例因不能耐受毒副反应而停止治疗。这也与以往研究相一致^[6, 7]^。

值得注意的是，几项临床研究CTONG0806^[13]^、TAILOR^[14]^和DELTA^[15]^的结果都告诉我们，与化疗相比，*EGFR*基因野生型患者无法从EGFR-TKI的治疗中获益。因此，在NSCLC患者的二线治疗时，首先应尽可能重新获取标本进行*EGFR*基因检测，再进行针对性治疗；本研究结果提示，如果在*EGFR*基因状态未明时，尤其是体质状况较差难以耐受化疗的患者可以考虑接受二线埃克替尼治疗。

## References

[1] Kim ST, Uhm JE, Lee J (2012). Randomized phase Ⅱ study of gefitinib versus erlotinib in patients with advanced non-small cell lung cancer who failed previous chemotherapy. Lung Cancer.

[2] Passaro A, Cortesi E, de Marinis F (2011). Second-line treatment of non-small-cell lung cancer: chemotherapy or tyrosine kinase inhibitors?. Expert Rev Anticancer Ther.

[3] Stinchcombe TE, Socinski MA (2009). Treatment paradigms for advanced stage non-small cell lung cancer in the era of multiple lines of therapy. J Thorac Oncol.

[4] Qi WX, Tang LN, He AN (2012). Effectiveness and safety of pemetrexed-based doublet versus pemetrexed alone as second-line treatment for advanced non-small-cell lung cancer: a systematic review and *meta*-analysis. J Cancer Res Clin Oncol.

[5] Qi WX, Shen Z, Yao Y (2012). *Meta*-analysis of docetaxel-based doublet versus docetaxel alone as second-line treatment for advanced non-small-cell lung cancer. Cancer Chemother Pharmacol.

[6] Kim ES, Hirsh V, Mok T (2008). Gefitinib versus docetaxel in previously treated non-small-cell lung cancer (INTEREST): a randomised phase Ⅲ trial. Lancet.

[7] Ciuleanu T, Stelmakh L, Cicenas S (2012). Efficacy and safety of erlotinib versus chemotherapy in second-line treatment of patients with advanced, non-small-cell lung cancer with poor prognosis (TITAN): a randomised multicentre, open-label, phase 3 study. Lancet Oncol.

[8] Wang HP, Zhang L, Wang YX (2011). Phase Ⅰ trial of icotinib, a novel epidermal growth factor receptor tyrosine kinase inhibitor, in Chinese patients with non-small cell lung cancer. Chin Med J (Engl).

[9] Shi Y, Zhang L, Liu X (2013). Icotinib versus gefitinib in previously treated advanced non-small-cell lung cancer (ICOGEN): a randomised, double-blind phase 3 non-inferiority trial. Lancet Oncol.

[10] Qi WX, Shen Z, Lin F (2012). Comparison of the efficacy and safety of EFGR tyrosine kinase inhibitor monotherapy with standard second-line chemotherapy in previously treated advanced non-small-cell lung cancer: a systematic review and *meta*-analysis. Asian Pac J Cancer Prev.

[11] Jiang J, Huang L, Liang X (2011). Gefitinib versus docetaxel in previously treated advanced non-small-cell lung cancer: a *meta*-analysis of randomized controlled trials. Acta Oncol.

[12] Chen JL, Liu XM, Wang LC (2014). Comparison of icotinib versus docetaxel as second-line treatment of advanced NSCLC. Shi Yong Zhong Liu Za Zhi.

[b13] 13Yang J, Cheng Y, Zhao M, *et al*. A phase Ⅱ trial comparing pemetrexed with gefitinib as the second-line treatment of nonsquamous NSCLC patients with wild-type *EGFR* (CTONG0806). J Clin Oncol, 2013, 31(suppl): abstr 8042.

[14] Garassino MC, Martelli O, Broggini M (2013). Erlotinib versus docetaxel as second-line treatment of patients with advanced non-small-cell lung cancer and wild-type *EGFR* tumours (TAILOR): a randomised controlled trial. Lancet Oncol.

[15] Kawaguchi T, Ando M, Asami K (2014). Randomized phase Ⅲ trial of erlotinib versus docetaxel as second- or third-line therapy in patients with advanced non-small-cell lung cancer: Docetaxel and Erlotinib Lung Cancer Trial (DELTA). J Clin Oncol.

